# Inhibition of choroidal neovascularization by anti-EphB4 monoclonal antibody

**DOI:** 10.3892/etm.2013.962

**Published:** 2013-02-18

**Authors:** DONGFENG SU, XIAOQIU LI, DIANWEN GAO

**Affiliations:** 1 Department of Ophthalmology, 463rd Hospital of Chinese People’s Liberation Army, Shenyang 110042;; 2 Department of Ophthalmology, Shengjing Hospital of China Medical University, Shenyang 110004;; 3 Department of Neurology, The General Hospital of Shenyang Military Region, Shenyang 110016, P.R. China

**Keywords:** EphB4, monoclonal antibody, choroidal neovascularization, progression

## Abstract

The aim of this study was to determine the effect of the EphB4 monoclonal antibody on experimental choroidal neovascularization (CNV) progression. Experimental CNV was established by argon laser photocoagulation. In the experimental group, the EphB4 monoclonal antibody was injected into the vitreous space in the eye specimens on days 0, 3, 6 and 9 after CNV model establishment. In the control group, an equal amount of balanced salt solution was injected at the same time points. On day 10 after CNV model establishment, fluorescein isothiocyanate-dextran endocardial perfusion and choroidal stretched preparation were conducted, respectively, for the two groups. The CNV area in each light spot and the mean values were determined. Histopathological examination was conducted and the ratio of the maximum thickness of the CNV in each light spot to the surrounding normal choroidal thickness, as well as the mean ratio, were calculated. Choroidal stretched preparation confirmed that the CNV of the experimental group was smaller, whereas the CNV of the control group was wider and larger. Quantitative analysis revealed that CNV in the experimental group was significantly inhibited (t=11.84, P<0.01) and that CNV progression in the experimental group was significantly suppressed (t=7.45, P<0.01). Histopathological examination revealed that CNV in the experimental group was thinner and smaller. Vitreous injection of the EphB4 monoclonal antibody inhibits experimental CNV progression. However, its specific mechanism remains unclear. Endogenous EphrinB2/EphB4 regulates ocular neovascularization and may become a new target in treating CNV diseases.

## Introduction

Choroidal neovascularization (CNV) is the main cause of macular diseases, including age-related macular degeneration (AMD), highly myopic maculopathy and impaired vision. The toughness of new vessels is extremely poor. These vessels usually break through Bruch’s membrane to enter the subretinal space, thereby causing exudation or hemorrhage, retinal tissue damage and rapid loss of vision, as well as inducing pigmentary epithelial detachment or retinal neuroepithelial detachment. CNV angiopoiesis is a series of complex pathological processes, including endothelial cell activation, extracellular matrix changes, basement membrane degradation, budding, proliferation and migration of endothelial cells, capillary loop formation and lumen transfixion. Although the angiopoiesis mechanism remains unclear, its processes are affected by a number of positive or negative regulation factors. Among them, the receptor tyrosine kinase (RTK) system is an important gene family and three vital RTKs are involved in CNV formation. They are the vascular endothelial growth factor (VEGF) and VEGF receptor (VEGFR), angiogenin/Tie receptor and Ephrin/Eph receptor systems. The VEGF/VEGFR system mainly induces vascular endothelial differentiation and angiopoiesis, whereas the angiogenin/Tie receptor system mainly regulates vascular maturation and quiescence in the later angiopoietic stages. The Ephrin/Eph receptor system affects the developing vascular endothelial cells and may play an important role in the neovascularization assembly process by transmitting a bidirectional signal, transferring position guide information and controlling asymmetric arteriovenous establishment (1).

The corresponding Ephrin ligand and Eph receptor expression in the neovascularization of ocular tissues, including the cornea, retina and choroid, as well as *in vitro* and *in vivo* animal experiments, all suggest that the Ephrin/Eph, VEGF/VEGFR and angiogenin/Tie receptor systems are all involved in the development and progression of ocular neovascularization. Although the VEGF/VEGFR system intervention has achieved a degree of efficacy in terms of its potential application value in the clinical treatment of relevant ocular neovascularization diseases (2,3), the results are not completely satisfactory. EphrinB2/EphB4 is the specific growth factor of vascular endothelial cells in the Ephrin/Eph family. EphrinB2 ligand and EphB4 receptor expression is definitely present in the *vasa sanguinea retinae* and choroidal vessels of rats, mice and cattle. Furthermore, EphrinB2/EphB4 expression is present in the retinal neovascularization model of high oxygen-induced retinopathy of mouse prematurity and the laser-induced CNV model, indicating that EphrinB2/EphB4 plays a role in retinal neovascularization and CNV formation (4,5).

In the present study, we applied argon laser photocoagulation to establish the experimental CNV model. The EphB4 monoclonal antibody was subsequently injected into the vitreous bodies in the eyes of experimental animals to observe its effect on experimental CNV progression and conduct a quantitative analysis investigating the regulation of the EphrinB2/EphB4 system on choroidal neovascularization. Thus, this study proposes a new model for the CNV angiopoiesis mechanism and provides a theoretical basis for the prevention and control of CNV diseases in the clinical setting.

## Materials and methods

### Animals

Healthy 7-week-old C57BL/6J female mice were provided by the Experimental Animal Center of the Military Medical Science Academy of the Chinese People’s Liberation Army. This study was carried out in strict accordance with the recommendations in the Guide for the Care and Use of Laboratory Animals of the National Institutes of Health. The animal use protocol was reviewed and approved by the Institutional Animal Care and Use Committee (IACUC) of the 463rd Hospital of Chinese People’s Liberation Army. The study was approved by the ethics committee of Shengjing Hospital of China Medical University, Shenyang, China.

### CNV model preparation

The experimental CNV model of C57BL/6J mouse eyes was established using the argon laser photocoagulation method. The experimental animals were anesthetized with 10% chloral hydrate solution (3.5 ml/kg body weight) by intraperitoneal injection. Mydrin-P and atropine gutta was used for mydriasis. Prior to surgery, the morphology of the fundus oculi was carefully observed in all experimental animals to confirm that it was normal. The argon laser was used with the following parameters: wavelength, 514 nm; light spot diameter, 100 *μ*m; shooting time, 0.1 sec and energy, 100 mW. In addition, the retinal posterior pole was arranged at the 9, 12, 3 and 6 o’clock positions around the optic disc to avoid the large *vasa sanguinea retinae* and each position was shot once at Bruch’s membrane breakthrough to induce experimental CNV. In case of laser excitation, the bubble generated at the photocoagulation position was regarded as the indicator of breaking through Bruch’s membrane.

Eighteen mouse eyes were randomly selected for the experimental group. On days 0, 3, 6 and 9 after CNV model establishment, 0.1 *μ*g 1 *μ*l EphB4 monoclonal antibody was injected into the vitreous space. The other 18 mouse eyes were classified as the control group. At the same time points, an equal amount of balanced salt solution was injected into the vitreous space. The specific method of vitreous injection was as follows: an experimental animal was anesthetized with 10% chloral hydrate solution (3.5 ml/kg body weight) by intraperitoneal injection and fixed. The upper and lower eyelids of the mouse were separated with ophthalmic microforceps. Orbital margin tissues were gently pressed to take out the eyeball. The needle of a 1 *μ*l microsyringe (Shanghai Anting Microsyringe Factory, Shanghai, China) was inserted at the position vertical to the eyeball wall at the rear of the superior temporal corneoscleral limbus and 1 *μ*l EphB4 monoclonal antibody or balanced salt solution was slowly injected. After removing the needle, the pinhole was gently and immediately pressed with a cotton swab to reset the eyeball. The eye was subsequently coated with erythromycin eye ointment.

On day 10 after CNV model establishment, high molecular weight fluorescein isothiocyanate (FITC)-dextran endocardial perfusion and choroidal stretched preparation examination were conducted on the experimental group and histopathological examination of the control group was performed to observe CNV progression. Quantitative analysis and comparison were conducted to evaluate the inhibition of the EphB4 monoclonal antibody on experimental CNV progression.

### FITC-dextran perfusion and choroidal stretched preparation examination

Thirty-six light spots of nine mouse eyes were assessed from the experimental and control groups. FITC-dextran perfusion and choroidal stretched preparation examination (1) was carried out according to the method described by Edelman and Castro (6). An experimental animal was anesthetized with 10% chloral hydrate solution (3.5 ml/kg) by intraperitoneal injection and fixed; then, the thoracic cavity was rapidly opened. Subsequently, 50 mg high molecular weight FITC-dextran (molecular weight, 2×10^6^; Sigma-Aldrich, St. Louis, MO, USA) was dissolved in 1.0 ml distilled water and injected into the left ventricle. At the late perfusion stages, the heart was gently pressed with the forceps to facilitate perfusion for adequate contrast. Next, the eyeball was removed and fixed in 4% formaldehyde. After 1–2 h, the eyeball was cut open along the corneoscleral limbus to remove the cornea, lens and retina and observe them under an operating microscope. The free choroid was sheared in a radial shape and spread onto a slide. After adding a small amount of glycerogelatin, the slide was covered with a cover slip. Finally, the choroidal stretched preparation was observed under a fluorescence microscope (Leica DM3000, Fukuoka, Japan) and photographed. At the same time, MacScope software (Mitani Co., Fukuoka, Japan) was used to calculate the experimental CNV area at each light spot and calculate the mean value of each group.

### Histopathological examination

Thirty-six light spots from mouse eyes were sampled from the experimental and control groups. After each animal was sacrificed, the eyeballs were removed, fixed in 4% formaldehyde for 24 h, conventionally dehydrated and embedded in paraffin wax. The optic nerve parallel to the sagittal plane at the laser photocoagulation position was selected and slices with a thickness of 6.0 *μ*m were prepared continuously. The sections were stained with hematoxylin-eosin, observed under a light microscope and photographed. At the same time, MacScope software was used to calculate the ratio (M/C) of the maximum thickness of experimental CNV in each light spot, that is, the distance from the choroidal bottom at the center of the light spot to the top of the neovascularization membrane (M) to the surrounding normal choroidal thickness (C). The mean values of the various groups were calculated.

## Results

### FITC-dextran perfusion and choroidal stretched preparation examination

**Figure 1:**
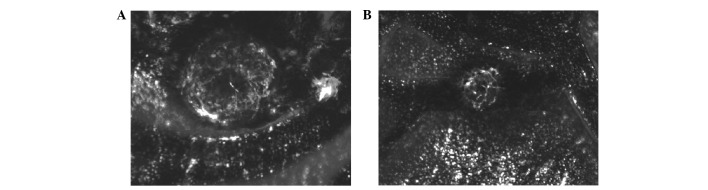
FITC-dextran perfusion and choroidal stretched preparation examination. (A) Choroidal stretched preparation 10 days after photocoagulation in the control group. CNV presented the reticular structure composed of flat vessels (magnification, ×100). (B) Choroidal stretched preparation 10 days after photocoagulation in the experimental group. The CNV area was smaller and there were fewer vessels. No reticular structure was formed (magnification, ×100). FITC, fluorescein isothiocyanate; CNV, choroidal neovascularization.

On day 10 following photocoagulation, CNV was observed in all light spots in the control group, presenting a larger reticular structure composed of flat vessels. In the experimental group, neovascularization network areas in the light spots were smaller, and a number only presented vascular circles. The quantitative analysis of the CNV area revealed that the mean CNV area of the control group was (28.12±3.79) ×10^−3^ mm^2^, whereas that of the experimental group was (19.19±2.48) ×10^−3^ mm^2^. The CNV of the experimental group was significantly inhibited (t=11.84, P<0.01; Fig. 1).

### Histopathological examination

**Figure 2:**
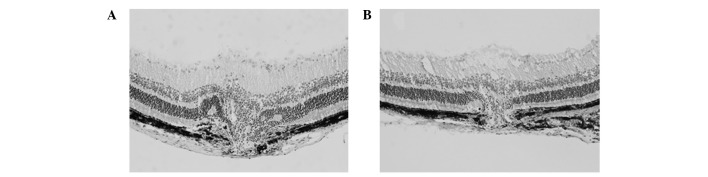
Histopathological examination. (A) On day 10 after photocoagulation in the control group, CNV presented vascular plexus with wide blood vessel lumen. It originated from the choroid and grew towards the bottom of the retina (HE; magnification, ×100); (B) On day 10 after photocoagulation in the experimental group, CNV was thinner and smaller and it grew towards the bottom of the retina (HE; magnification, ×100). CNV, choroidal neovascularization; HE, hematoxylin and eosin.

In the experimental group, CNV was thinner and smaller. Quantitative analysis of the CNV thickness revealed that the ratio (M/C) of the control group was 2.60±0.63, whereas that of the experimental group was 1.74±0.28. CNV progression in the experimental group was significantly inhibited (t=7.45, P<0.01; Fig. 2).

## Discussion

The Eph receptor and Ephrin ligand are divided into subclasses A and B according to the sequence conservation and their affinity difference. Nine types of EphA receptors (EphA1 to A9), 6 types of EphrinA ligands (EphrinA1 to A6), 6 types of EphB receptors (EphB1 to B6) and 3 types of EphrinB ligands (EphrinB1 to B3) have been identified (7). EphrinA is attached to the cell membrane via glycosyl-phosphatidyl inositol (GPI), whereas EphrinB is a transmembrane protein. The characteristics of Ephrin ligands depend on the requirement to bind and break through the cell membrane and thereby activate the function of corresponding receptors. Therefore, the Ephrin/Eph interaction depends on direct intercellular contact (8). Unlike other RTKs, the Eph receptor and ligand are subject to phosphorylation, thereby mediating bidirectional signal transmission.

In cultured endothelial cells, typical positive signals (EphrinB2 to EphB4) reduce the proliferation and migration of the EphB4 cell. On the contrary, negative signals (EphB4 to EphrinB2) increase the proliferation and migration of the EphrinB2 cell (9–16). Such bidirectional effects of EphrinB2/EphB4 may regulate the endothelial cell guidance and spatial combination process and thus cause the correct differentiation of arteriovenous vessels, as well as the reasonable division of arterial and venous capillaries. EphrinB2 and its receptor, EphB4, are respectively regarded as relatively specific molecular markers of the original artery and original vein and they specifically damage the signal transduction of EphrinB2/EphB4, which causes remodeling defects and developmental defects in the primary capillary network of the embryonic vascular system, thereby resulting in embryonic mortality (17–21).

He *et al* (5) observed EphB4 and EphrinB2 expression in the cultured choroidal capillary endothelial cells of rats and the laser-induced CNV membrane, in which EphB4 expression was stronger than EphrinB2 expression. EphB4 dominated in physiological or pathological choroidal vessels. Erber *et al* (22) identified that the EphB4 pathway plays an important role in the regulation process of acquired vascular configuration remodeling, permeability and other aspects, including tumors and ocular vascular tissues. This function is independent of EphB4 protein tyrosine kinase receptor activity, namely the non-positive EphB4 pathway. EphB4 participates in the above process by binding with the ligand EphrinB2 in the negative signal pathway. He *et al* (5) administered an intravitreous injection of the soluble monomer sEphB4 *in vitro* and identified that sEphB4 inhibits laser-induced CNV development in rats and that the neovascularization area and permeability are greatly reduced. Another study (4) reported that the soluble chimera EphB4-Fc (artificially synthesized EphB4 extracellular domain composition bound with an immunoglobulin Fc fragment to form the chimera with a dimerization effect; used as an EphrinB2 agonist) also inhibits CNV formation. Contrastingly, the findings based on tumors revealed that EphB4 is located on the tumor cell surface. After the EphB4 extracellular domain is bound with vascular EphrinB2, it promotes angiopoiesis and endothelial cell migration and proliferation, thereby promoting tumor growth; whereas soluble sEphB4 inhibits tumor growth and angiopoiesis (23). These results are contrary to the negative signal theory; however, the specific mechanism is unclear. A number of scholars consider that the ‘bidirectional signal’ theory is based on *in vitro* cultured cells and is affected by numerous factors. Therefore, it cannot be simply copied *in vivo.*

In the present study, EphB4 monoclonal antibody was injected into the vitreous space to specifically damage the EphrinB2/EphB4 signal transduction. The results demonstrated that EphB4 significantly inhibited argon laser-induced CNV in mouse eyes. Compared with the control group, the CNV area and the ratio of maximum thickness of neovascularization to normal choroidal thickness in the experimental group were significantly reduced. We consider that after the EphB4 monoclonal antibody specifically binds with the EphB4 receptor, it blocks the negative signal pathway to the maximum extent and inhibits the neovascularization configuration remodeling, thus suppressing vascular development. In addition, the EphB4 monoclonal antibody interferes with the functions of other vascular factors by blocking EphrinB2/EphB4 signal transduction.

Similar to other RTKs, including VEGF and basic fibroblast growth factor (bFGF), Eph-Ephrin has a conserved tyrosine residue. However, the possibility of crosswise binding and transmitting signals between them remains unclear. Few reports are available on the correlation of VEGF with an Eph receptor and its Ephrin ligand. One study suggested that VEGF induces EphrinB2 expression and activates the potential EphB4 receptor (21). However, after the Eph receptor binds with the ligand, it weakens the cell growth induced by growth factors, including VEGF and bFGF. In the present study, CNV growth was inhibited by blocking endogenous EphrinB2/EphB4 signal transduction, which was not consistent with the above theory. The possible reason lies with the involvement of CNV formation in the joint regulation of a number of pathways and multiple cytokines, which requires further investigation.

The role of EphrinB2/EphB4 signal transduction in angiopoiesis has been increasingly studied. One study (24) proposed the feasibility of treating human malignancy- and angiopoiesis-related diseases by humanized low-immunity rat EphB4 monoclonal antibody. Another study observed EphB4 receptor expression in human iris tissue and cultured vascular endothelial cells of the iris (25). Furthermore, EphB4 receptor expression exists in human choroidal tissue. Therefore, the EphB4 monoclonal antibody is expected to become a promising treatment in the intervention treatment of CNV. In the present study, EphB4 monoclonal antibody was unable to inhibit neovascularization formation completely. Combined with the achieved results for the interventional treatment of CNV targeting VEGF/VEGFR, the successful treatment of targeting CNV formation should begin simultaneously with a number of pathways and multiple cytokines.
